# Systematic Review and Meta-analysis of Lyme Disease Data and Seropositivity for *Borrelia burgdorferi*, China, 2005‒2020

**DOI:** 10.3201/eid2812.212612

**Published:** 2022-12

**Authors:** James H. Stark, Xiuyan Li, Ji Chun Zhang, Leah Burn, Srinivas R. Valluri, Jiaxin Liang, Kaijie Pan, Mark A. Fletcher, Raphael Simon, Luis Jodar, Bradford D. Gessner

**Affiliations:** Pfizer Inc., Collegeville, Pennsylvania, USA (J.H. Stark, K. Pan, L. Jodar, B.D. Gessner);; Yale School of Public Health, New Haven, Connecticut, USA (X. Li);; Pfizer China, Beijing, China (J.C. Zhang, J. Liang);; P95 Pharmacovigilance & Epidemiology, Princeton, New Jersey, USA (L. Burn);; Pfizer Inc., New York, New York, USA (S.R. Valluri);; Pfizer Emerging Markets, Paris, France (M.A. Fletcher);; Pfizer Vaccine Research & Development, Pearl River, New York, USA (R. Simon)

**Keywords:** Lyme disease, Borrelia burgdorferi, tick-borne diseases, vector-borne infections, epidemiology, seroepidemiologic studies, bacteria, parasites, China

## Abstract

Since its initial identification in 1986, Lyme disease has been clinically diagnosed in 29 provinces in China; however, national incidence data are lacking. To summarize Lyme disease seropositivity data among persons across China, we conducted a systematic literature review of Chinese- and English-language journal articles published during 2005‒2020. According to 72 estimates that measured IgG by using a diagnostic enzyme-linked assay (EIA) alone, the seropositivity point prevalence with a fixed-effects model was 9.1%. A more conservative 2-tier testing approach of EIA plus a confirmatory Western immunoblot (16 estimates) yielded seropositivity 1.8%. Seropositivity by EIA for high-risk exposure populations was 10.0% and for low-risk exposure populations was 4.5%; seropositivity was highest in the northeastern and western provinces. Our analysis confirms Lyme disease prevalence, measured by seropositivity, in many Chinese provinces and populations at risk. This information can be used to focus prevention measures in provinces where seropositivity is high.

Lyme disease is a tickborne zoonosis caused by genospecies of *Borrelia burgdorferi* sensu lato that occur primarily in the Northern Hemisphere, including North America, Europe, and some countries in Asia (*1*). In China, Lyme disease has been an emerging disease since the first human case was documented in Heilongjiang Province in 1986 (*2*). Multiple genospecies of *B. burgorferi* have been identified in China, although only *B. garinii*, *B. afzelii*, and *B. valaisiana*–related genospecies have been reported to cause disease in humans (*3*,*4*).

*B. burgorferi* is transmitted to humans by *Ixodes* ticks and in China specifically by *I. persulcatus*, *I. sinensis*, and *I. granulatus* ticks (*5*–*7*). Of those species, *I. persulcatus* ticks are regarded as the most competent vectors and are frequently identified in northeastern and select western, central, and eastern provinces (*6*). Lyme disease is widely distributed across China, and cases have been documented in 29 provinces across the country, several of which show endemicity in certain regions, specifically the northeastern provinces (*5*).

During the past several decades, Lyme disease has emerged as a public health issue for China; however, lack of information about disease burden makes it difficult for national and local governments to effectively develop and implement prevention strategies. No national Lyme disease surveillance exists in China, and no estimates of national disease incidence have been published. Thus, the only available approach for quantifying disease risk is human *B. burgorferi* seroprevalence, which reflects the proportion of persons in the population with positive serum test results for the pathogen. During 1987–1996, seroprevalence summarized from 22 provinces indicated an average seropositivity rate of 5.06% (*8*). Most of those early investigations focused on persons employed in forestry and were geographically limited to the northeastern provinces. Subsequently, human seropositivity data have been reported for provinces across all of China: in populations for which tick exposure varies, in populations in different occupations and age groups, and by using different diagnostic testing approaches (*9*).

To summarize published human Lyme disease seropositivity data for 2005–2020, we reviewed data from the literature. We provide updated summary estimates of seropositivity for individual exposure risk, by distinct provinces and for China overall, based on diagnostic testing approaches to determine exposure to *B. burgorferi*.

## Methods

### Search Strategy and Selection Criteria

We conducted a global systematic literature review across 5 databases, following the Preferred Reporting Items for Systematic Reviews and Meta-Analyses (PRISMA) guidelines (*10*). We tailored the search to each database accessed: PubMed, EMBASE, CABI direct, China National Knowledge Infrastructure, and Wanfang Data (Appendix Table 1); we limited the search to articles published from January 1, 2005, through December 31, 2020. After performing the keyword search and reviewing the abstracts of retained articles, we assessed full-text articles to confirm their eligibility for inclusion. Articles were included only if they reported numerator (clearly indicating the number of seropositive persons) and denominator (the population tested) and had a diagnostic testing strategy that included an enzyme immunoassay (enzyme-linked assay [EIA] or ELISA), immunofluorescence assay (IFA), or Western immunoblot (WB). We excluded articles that did not describe the sample population for the study. We used a snowball technique to identify additional eligible articles in the reference lists of excluded literature review articles.

The protocol for the English-language literature review was published in the PROSPERO database (registration CRD42021236906, https://www.crd.york.ac.uk/prospero/display_record.php?ID=CRD42021236906). The search and extraction of the Chinese-language databases (China National Knowledge Infrastructure and Wanfang Data) occurred independently of the English-language literature review.

### Variables

In China, human serum is analyzed for the presence of *Borrelia*-specific IgM or IgG with either an EIA (or ELISA) or an IFA (*11*). If the EIA or IFA result is positive or equivocal, a more specific WB (or line blot) is subsequently conducted; this method is referred to as a standard 2-tier testing approach. This approach emphasizes sensitivity initially with the first-tier test and then with the second-tier test (*12*). However, this approach is not consistently used in China (*13*). In general, diagnostic assays were not well characterized in many of the included articles because there was limited information on diagnostic performance data, standardization criteria for all genospecies, and consistency in assay specifications (e.g., antigens and reagents used).

The primary analytical strategy prioritized IgG measurements based on a single-tier EIA or IFA test. Although IgM-based tests are useful for clinical diagnosis of an early infection, they are also more likely than IgG-based tests to yield false-positive results; consequently, a sensitivity analysis was conducted for seropositivity estimates derived from an EIA or IFA that did not distinguish the results as either IgM or IgG positive. A second diagnostic sensitivity analysis was performed for estimates reporting 2-tier testing (EIA or IFA followed by WB), which may serve as a truer indicator of seropositivity. Neither sensitivity analysis included estimates used for the primary analytical strategy. We conducted subgroup analyses for estimates reported by exposure, sex, age group, and province based on an IgG measurement as determined by an EIA or IFA, similar to the primary analysis. 

To adequately reflect potential variation in exposure to ticks and transmission of *Borrelia*, we characterized exposed populations. The study populations within reviewed articles were categorized into 2 broad categories: by clinical suspicion (sample identified from hospital or clinic settings, which is an unknown reflection of risk) or by exposure risk (risk for exposure to natural foci of Lyme disease, either by location or by occupation). Clinical suspicion cases are identified in hospital or clinic settings from persons with a history of suspected tick bites or with a clinical suspicion of Lyme disease (e.g., arthritis, nervous system disease, or early symptoms). To reduce biasing the risk assessment, we assessed exposure risk groups before performing statistical analyses. We categorized low exposure risk as persons who worked or lived in either nonforested plains areas or urban environments or who had minimal or no exposure to tick-infested habitats, medium exposure risk as persons whose work or location exposed them to tick-infested habitats but whose exposure was neither frequent nor prolonged, and high exposure risk as persons whose work or location frequently exposed them to forested areas or other areas where prolonged exposure to tick-infested habitat might have occurred.

### Statistical Analyses

We descriptively summarized all articles for this meta-analysis and calculated fixed-effects summary estimates. Although the reviewed studies may be sufficient for drawing conclusions about the relationship between exposure and the outcome from the fixed-effects model, the studies themselves could be highly variable. Therefore, we conducted tests of homogeneity for the study samples for all studies. We assumed the variable “province” to be random and re-evaluated seropositivity to assess the robustness of the estimates by using the mixed-effects model. We considered a fixed-effects meta-analysis to be an appropriate method for summarizing seropositivity data for Lyme disease as the primary analytical strategy for the sensitivity analyses.

We used the number of available seropositivity estimates to calculate the overall least square mean summary estimate, SE, and lower and upper 95% CIs by using PROC Mixed in SAS (SAS Institute Inc., https://www.sas.com), in which the response term was the outcome (seropositivity) and the class term was province. We developed odds ratios to estimate the odds of an association between high-exposure risk group seropositivity and low-exposure risk group seropositivity, including corresponding 95% CIs. Given the paucity of data and variables available from each article, we made no adjustment for confounding in the fixed-effects models. We used forest plots to display the distribution of seropositivity and heterogeneity of the summarized seropositivity results. All analyses were conducted by using SAS version 9.4.

## Results

**Figure 1:**
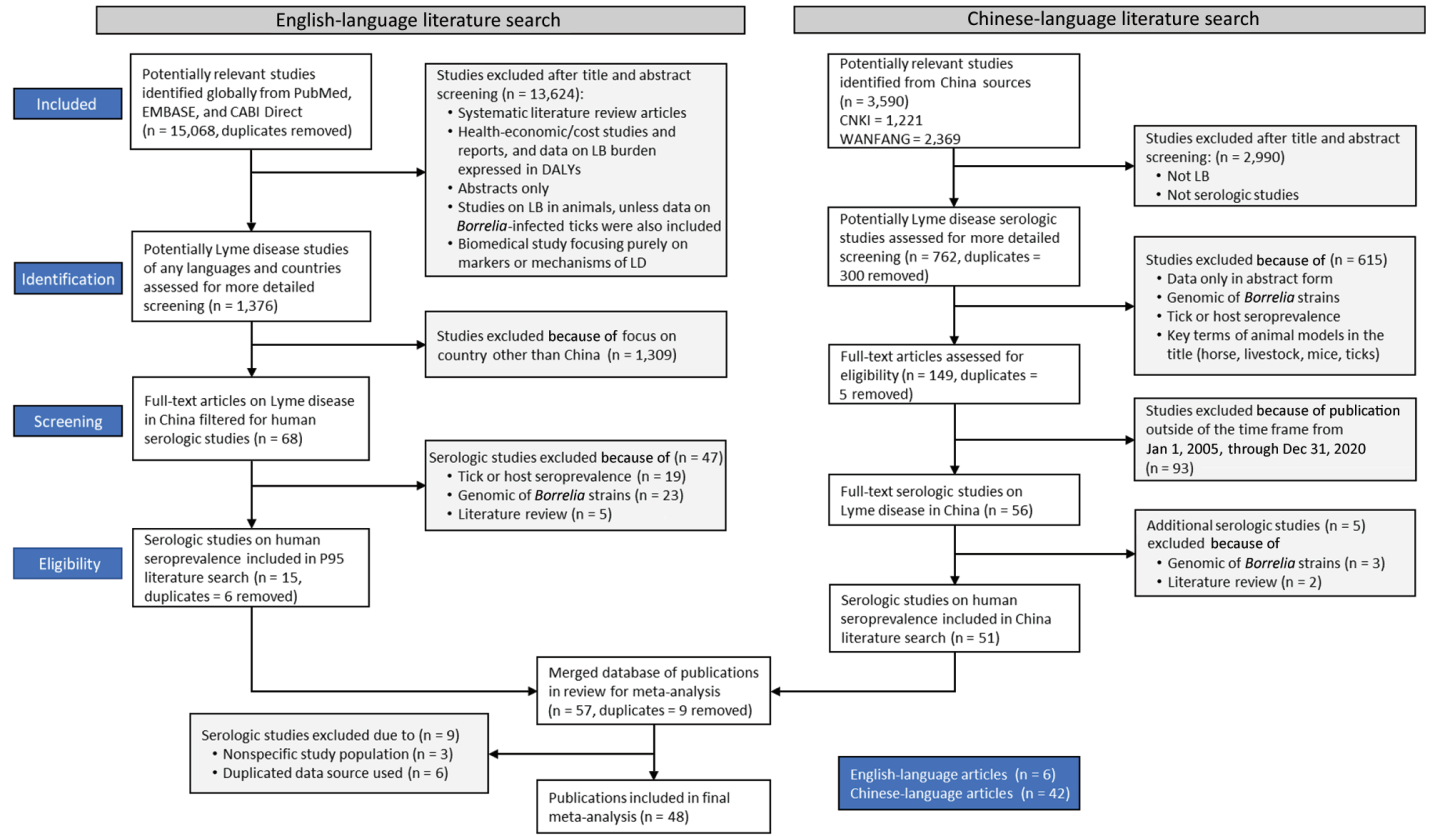
Preferred Reporting Items for Systematic Reviews and Meta-Analyses (PRISMA) (*10*) flow diagram of the 2 literature searches performed in review of seropositivity for *Borrelia burgdorferi* in China, 2005–2020. DALY, daily adjusted life years; LB, Lyme borreliosis; LD, Lyme disease.

**Figure 2:**
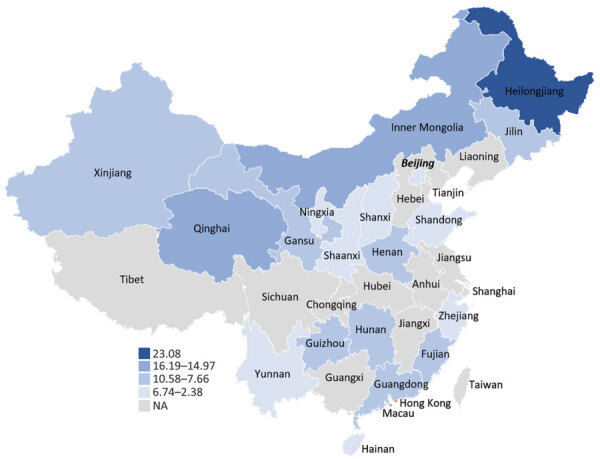
Estimated seropositivity for *Borrelia burgdorferi,* by province, China, 2005–2020. *Ixodes persulcatus* ticks, among the most frequently identified ticks in China, have been found across the northeastern and select western, central and eastern provinces. *I. sinensis* and *I. granulatus* ticks are the main identified vectors in the southern and eastern regions of the country. Variations in seropositivity reflect differences in tick competency, tick bite risk, and diagnostic tests. Numbers in key are percentages. NA, not applicable.

**Figure 3:**
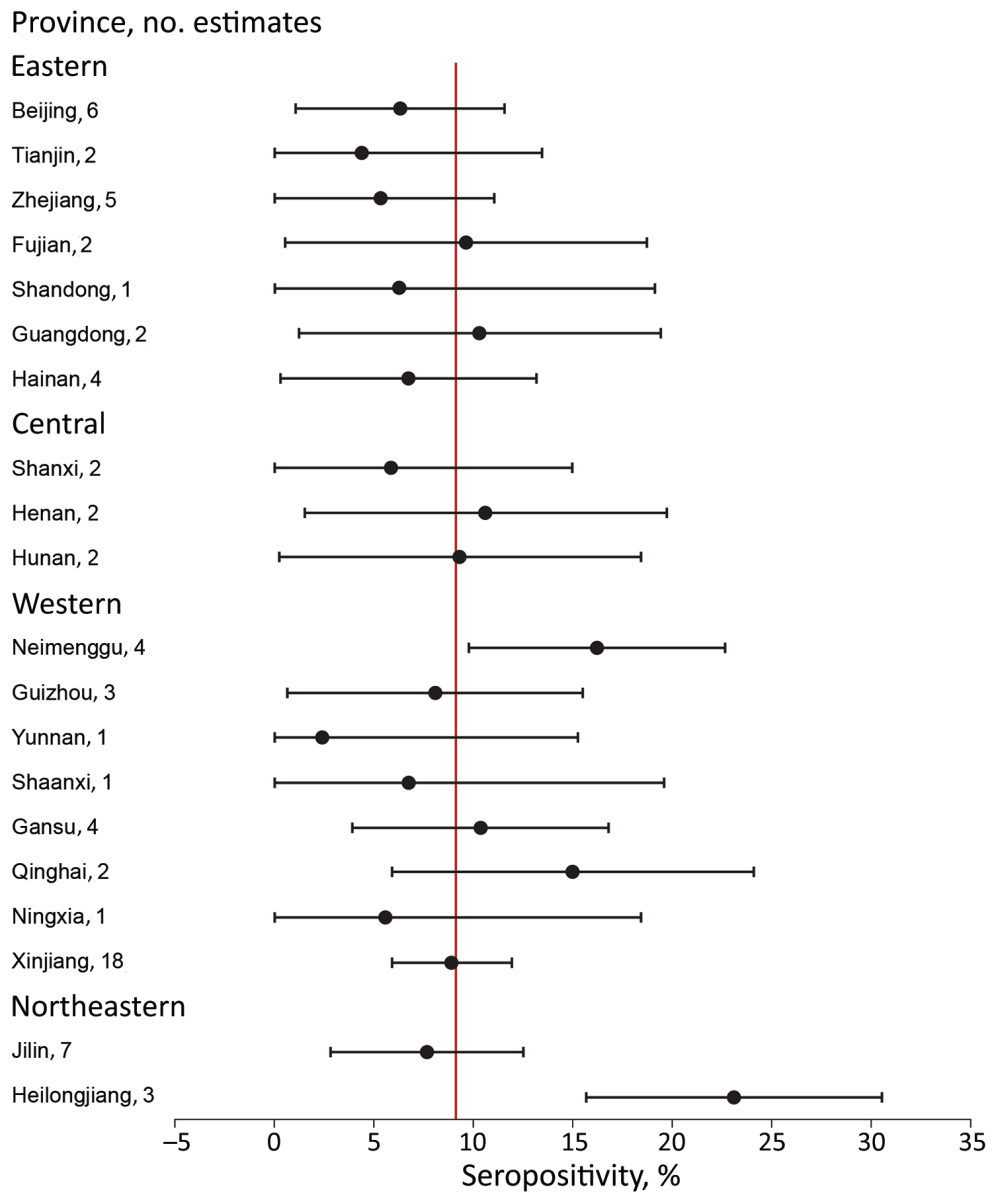
Forest plot illustrating seropositivity estimates for *Borrelia burgdorferi,* by province, China, 2005–2020. The red horizontal line indicates the summary estimate based on the primary analysis; error bars indicate 95% CIs. For 7 estimates, the lower bound of the 95% CI was <0 (a negative value); those values were fixed at 0% for interpretation.

Our literature review identified 3,657 articles that focused on China; 48 articles met the selection criteria (Figure 1), of which 42 articles met the criteria for the primary analytical strategy. In total, these 42 articles provided 72 estimates of seropositivity that we extracted for analysis. Some articles produced seropositivity estimates for multiple provinces or years (Appendix Table 2). Six articles did not meet the criteria for the primary analytical strategy; thus, they contributed data to only the 2 diagnostic sensitivity analyses. From the included studies, we compiled a description of estimates by subgroup (Table 1), by exposure group and province (Table 2), and by province (Figures 2, 3).

**Table 1:** Modeled estimates of seropositivity for *Borrelia burgdorferi* sensu lato in China, 2005–2020*

Variable	Seropositivity estimates, no. (study denominator sample size)†	Modeled seropositivity, % (95% CI)
Primary analysis: IgG only	72 (34,719)	9.1 (7.5–10.7)
Sensitivity analysis		
IgM and IgG	35 (9,446)	14.5 (11.8–17.2)
EIA‡ + WB	16 (8,837)	1.8 (0.9–2.7)
Exposure group		
Clinical suspicion	10 (3,982)	7.1 (6.4–8.0)
Low risk	10 (5,245)	4.5 (3.9–5.1)
Moderate risk	12 (5,300)	6.1 (5.4–6.7)
High risk	40 (20,192)	10.0 (9.6–10.4)
Sex		
F	21 (7,542)	10.0 (6.6–13.2)
M	21 (8,223)	9.4 (6.2–12.6)
Age group, y		
<20	13 (1,420)	12.0 (4.4–19.6)
20–29	11 (1,416)	12.3 (6.3–18.4)
30–39	11 (1,734)	14.5 (5.9–23.1)
40–49	11 (1,757)	14.2 (8.5–20.0)
50–59	11 (1,434)	13.1 (8.5–17.7)
>60	12 (1,429)	12.6 (6.6–18.5)

*EIA, enzyme immunoassay; WB, Western blot. †Positive test results: primary analysis = 2,859; sensitivity analysis IgM and IgG = 1,260; sensitivity analysis EIA + WB = 147. ‡First-tier test was either an ELISA or immunofluorescence assay.

**Table 2:** Distribution of estimates of seropositivity for *Borrelia burgdorferi* sensu lato*,* by exposure group and province, China, 2005–2020

Province	Total no. estimates, n = 72	Clinical suspicion estimates, n = 10	Exposure group		
			Low risk, n = 10	Moderate risk, n = 12	High risk, n = 40
Beijing	6	2	1	1	2
Fujian	2	0	1	0	1
Gansu	4	0	0	0	4
Guangdong	2	0	0	1	1
Guizhou	3	0	1	1	1
Hainan	4	4	0	0	0
Heilongjiang	3	2	0	0	1
Henan	2	0	0	0	2
Hunan	2	0	0	0	2
Jilin	7	0	1	1	5
Neimenggu	4	1	1	0	2
Ningxia	1	0	0	0	1
Qinghai	2	0	0	0	2
Shaanxi	1	0	0	0	1
Shandong	1	0	0	0	1
Shanxi	2	0	0	0	2
Tianjin	2	0	1	0	1
Xinjiang	18	0	2	8	8
Yunnan	1	0	1	0	0
Zhejiang	5	1	1	0	3

For the primary analytical strategy, the reported IgG seropositivity estimates based on a single-tier test (EIA or IFA) ranged from 0 to 37%; the fixed-effects modeled summary estimate was 9.1% (95% CI 7.5%–10.7%) (Table 1). When the random-effects model was used for the primary analytical strategy, neither estimate nor variance differed. The total sample size producing this summary estimate was 34,719 (Appendix Table 2). 

Fewer articles and estimates were available for the diagnostic sensitivity analyses. For the sensitivity analysis based on 35 estimates (sample size of 9,446 obtained from 5 articles) that did not distinguish between IgG and IgM results, seropositivity was 14.5% (95% CI 11.8%–17.2%). For the sensitivity analysis that used a 2-tier testing system (16 estimates obtained from 6 articles with a sample size of 8,837), seropositivity was 1.8% (95% CI 0.9%–2.7%) (Table 1).

Seropositivity for the clinical suspicion sample was 7.1% (95% CI 6.4%–8.0%). Lyme disease seropositivity estimates by exposure risk populations were 4.5% (95% CI 3.9%–5.1%) for low risk, 6.1% (95% CI 5.4%–6.7%) for medium risk, and 10.0% (95% CI 9.6%–10.4%) for high risk (Table 1). The odds ratio of high exposure risk seropositivity compared with low exposure risk seropositivity was 2.4 (95% CI 2.1–2.7) and of moderate exposure risk seropositivity compared with low exposure risk seropositivity was 1.4 (95% CI 1.2–1.6).

Variation by province was substantial; the highest seropositivity estimates were 23.1% for Heilongjiang Province and 16.2% for Neimenggu (Inner Mongolia) Province (Figure 2). Moreover, variation across provinces was substantial (Figure 3). There was no discernable trend over time for seropositivity (data not shown).

## Discussion

Our systematic literature review of *B. burgdorferi* seropositivity in China generated summary estimates by diagnostic test, exposure risk, sex, age group, province, and year. Depending on the testing algorithm applied, the seropositivity ranged from 1.8% to 14.5%, reflecting Lyme disease endemicity in the population. Combined with the widespread distribution of *Ixodes* ticks, specifically *I. persulcatus* ticks in many provinces, this analysis reinforces that Lyme disease is a public health problem in China.

The summary estimates of 9.1% among EIA/IFA positive samples and 1.8% among samples confirmed with WB fell within the range identified in Europe. In Germany, the nationwide, population-based cross-sectional KiGGS study estimated seropositivity among children and adolescents of 4.8% by single-tier ELISA testing (4% when confirmed by line blot) (*14*). A similar nationwide, population-based, cross-sectional study among adults in Germany (DEGS) reported overall seropositivity of 9.4%, confirmed by line blot (*15*). A cross-sectional health survey among a representative sample of adults in Finland reported seropositivity of 3.9% according to 2-tier testing (*16*). A representative sample of healthy blood donors from the Tyrol region of Austria reported a seropositivity range of 1.5%–7.2% from samples confirmed by line blot (*17*). A regional study in Turkey among healthy volunteers revealed seropositivity of 4.1% by single-tier testing with ELISA and 2.2% confirmed by WB (*18*).

In China, results of the diagnostic sensitivity analyses were consistent with expectations based on Lyme diagnostic testing limitations. Several studies did not adequately delineate the results by IgM or IgG positivity, and this joint numerator resulted in a substantially higher estimate than IgG seropositivity alone. IgM responses to *Borrelia* may persist over time, although IgM reactivity alone without isotype switching to IgG may reflect a false-positive result (*12*,*19*). False-positive results may provide an explanation for the higher seropositivity found when including results that did not distinguish between IgM and IgG. Testing results were further complicated in many studies by assays that used a whole-cell sonicate; such a lysate generates multiple antigens that can increase the likelihood that cross-reactive IgG or IgM creates a false-positive result compared with newer EIAs that focus on a reduced set of well-defined purified antigens specific to *B. burgdorferi* genospecies (*12*).

In many countries in Europe and in the United States, a 2-tier testing system for Lyme disease is used for clinical diagnosis and seroprevalence assessments. The sensitive first-tier test uses an ELISA, or less often an IFA, followed by a highly specific, second-tier WB if the ELISA is positive or equivocal. Diagnostic sensitivity can vary widely, with estimates ranging from as low as 14% during the early stages of disease to 100% as symptoms and manifestations evolve (*12*). The value of this standard 2-tier testing approach is improved specificity compared with ELISA or IFA alone (*9*,*20*). Specificity in all clinical phases is robust at >99% after the second-tier test. Recently, a modified 2-tier testing system based on 2 EIAs, which substitutes an EIA for the second-tier WB, has been implemented. Third-generation EIAs focus on select antigens; as a result, pairing with different EIAs enables substantial orthogonality that improves sensitivity while maintaining specificity (*12*). Neither of those 2-tier testing approaches has been widely adopted in China (*13*), where studies reporting the seropositivity of antibodies to *B. burgdorferi* relied primarily on the first-tier EIA or IFA and less often on the confirmatory, specific WB. 

Furthermore, diagnostic performance data are not readily available from China, particularly across the range of testing procedures used. Some studies acknowledged use of reagents provided by the China Centers for Disease Control and Prevention, others used local commercial test kits, a few used nondomestic test kits, and several others did not describe the assays used. The China Centers for Disease Control and Prevention described criteria for standardization of a WB based on lysates of *B. garinii* bacteria; the assay used a single band for IgG or IgM, a criterion that differs from guidelines in Europe and the United States that require at least 2 of 3 bands by IgM or 5 of 10 bands by IgG to be classified as positive by WB (*12*,*13*). In addition, interpretation criteria were not consistent across associated articles. The few articles from our review that reported seropositivity with a confirmatory WB result led to a summary estimate substantially lower than the single-tier test (1.8% vs. 9.1%). The limited information on quality of assays, accreditation, and validation remains a major limitation of analytic interpretability. Nevertheless, the sampling for the 2-tier testing strategy occurred in 4 provinces across all types of exposure categories, providing some evidence of a representative seroprevalence estimate (Appendix Table 2).

Seropositivity was higher for populations that had been assessed as having a higher risk for exposure to a natural foci of Lyme disease, either by occupation or by location. These data were consistent with targeted samples of higher risk occupational groups from other countries. For instance, IgG seropositivity among farmers was 5.5%–9.7% in Belgium (*21*) and 10%–13.7% in Poland (*22*,*23*). Forestry workers are among the most sampled high exposure risk occupational groups; reported seropositivity was 14% in Lithuania (*24*), 31% in Hungary (*25*), 7.8% in Italy (*26*), 11.8% in Serbia (*27*), 10.9% in Turkey (*28*), 21.6% in Belgium (*29*), and 14%–34% in Poland (*22*,*30*). Likewise, increased seropositivity seen in medium to high exposure risk populations in China and consistency with data from Europe provide initial confidence in the overall estimates provided in this report.

The summary estimate in the clinical suspicion sample, which reflected a mixture of studies focused on history of a suspected tick bite or clinical suspicion of Lyme disease, was 7.1%. The TBD STING study in Sweden and Finland reported seroconversion after a tick bite for 3.5% of participants (*31*). Nonetheless, seroconversion does not necessarily reflect clinical infection because Lyme disease manifestation during the 3-month follow-up period did not develop for 57.6% of the *Borrelia*-infected persons who seroconverted. Among the seroconversions that resulted in clinical manifestations, these included erythema migrans (85%), *Borrelia* lymphocytoma (3%), nervous system disease (6%), or nonspecific symptoms of Lyme disease (6%). A study conducted in China documenting clinical manifestations after tick bite reported a lower proportion of erythema migrans (69%) and a higher proportion of nervous system disease (21%) and arthralgia (21%), among other manifestations (*32*). In the United States, a randomized controlled trial of a vaccine candidate documented an asymptomatic proportion of <10%, potentially arising from the shorter duration of follow-up for symptoms compared with that in Europe (*33*). Notwithstanding, differences in asymptomatic proportions and clinical manifestations between studies and countries may reflect differences in study design, duration of follow-up, diagnostic quality, timing of postinfection treatment, antibody waning, and circulating genospecies.

The highest summary seropositivity estimates were detected in 2 provinces in northeastern China (Heilongjiang Province, 23.1%) and western China (Neimenggu Province [Inner Mongolia], 16.2%). *I. persulcatus* ticks are among the most frequently identified ticks in China and have been found across the northeastern and select western, central, and eastern, provinces (*6*). Several other provinces that border Heilongjiang and Neimenggu Provinces have frequently reported the presence of *I. persulcatus* ticks, although the reported seropositivity has been lower (7%–10%). The lower calculated estimates within these provinces could reflect differences in the sampled exposure groups because studies from Heilongjiang and Neimenggu Provinces largely focused on populations for which higher infection prevalence was expected (e.g., forest residents and forestry workers). In addition, no samples from Heilongjiang and Neimenggu Provinces were tested by using the more conservative 2-tier testing strategy, which probably would have resulted in lower seropositivity estimates. Alternatively, lower seropositivity in border regions could reflect true differences in exposure risk. Other regions of China, particularly those in the southern and eastern regions, reported somewhat lower seropositivity (2%–10%). The distribution of *I. persulcatus* ticks is limited in these regions, although *I.* *sinensis* and *I. granulatus* ticks have been reported and are considered to be the main vectors in these provinces. However, demonstration of vector competency and efficiency of *I. sinensis* and *I. granulatus* ticks as vectors of *B. burgorferi* is unclear (*5*,*7*).

Other caveats to consider include the possibility that seropositivity may be driven by persons who become infected in higher incidence regions but reside in regions without efficient local transmission, because their limited awareness of Lyme disease precludes appropriate personal prevention measures. Another consideration is the local prevalence of Lyme disease for such persons. Despite good specificity of the test, a low a priori probability of disease will lead to a lower positive predictive value for true disease (*12*). Regardless of these caveats, increasing tick distributions across China has been attributed to planned reforestation and changing land use patterns leading to suitable environments to maintain the tick enzootic cycle and ultimately *Borrelia* transmission (*3*).

Among the study limitations, there were substantial variations in populations sampled (including persons seeking clinical care, or convenience samples), in risk exposure population targeting, and in varying study designs (none of the studies were designed to be nationally representative). More than half of the studies were conducted among a higher risk exposure population that probably elevated the summary estimate. With additional information on the percentage of the country’s population at different levels of risk, a weighted average could be obtained, although this type of data is difficult to quantify. Second, the included studies used a range of testing methods that may not be comparable. For example, IFA was widely used to estimate seropositivity, but this traditional, manual method relies on the experience of the technician, leading to potentially lower specificity compared with enzyme immunoassay (EIA or ELISA) (*9*,*20*). In addition, 2-tier testing was not uniformly used, which could have resulted in potentially higher rates of false-positive results. Third, exposure risk may have been misclassified because an accurate description of specific testing populations may be missing from source manuscripts. Fourth, although *B. garinii* and *B. afzeli* have been reported as the predominant circulating genospecies, other circulating nonpathogenic genospecies may cause a false-positive result (*3*,*4*). Last, seropositivity estimates reflect exposure risk for infection regardless of clinically apparent disease; therefore, these summary estimates should not be interpreted as reflecting risk for clinical disease. Collectively, these limitations portend to overestimate seropositivity compared with other results as noted in Europe.

In conclusion, the results from this meta-analysis demonstrate seropositivity to *B. burgorferi* in China over the past several decades, particularly in certain provinces and in high exposure risk populations. By itself, however, the utility of this information for driving public health policy is limited because it gives no indication of clinical burden, either overall or by severity, and may not accurately represent geographic variations in risk. The expanding geographic range of infected ticks and increased likelihood of contact with humans will continue to present a public health challenge for China.

Appendix Additional information for systematic review and meta-analysis of Lyme disease data and seropositivity for *Borrelia burgdorferi*, China, 2005‒2020.
