# Supplementary material for: Systematic Review and Meta-analysis of Lyme Disease Data and Seropositivity for Borrelia burgdorferi, China, 2005‒2020
Source: Emerg Infect Dis. 2022 Dec;28(12):2389–97. doi: 10.3201/eid2812.212612 (PMC9707590; doi:10.3201/eid2812.212612)
Supplement: Appendix — Additional information for systematic review and meta-analysis of Lyme disease data and seropositivity for Borrelia burgdorferi, China, 2005‒2020. [file 21-2612-Techapp-s1.pdf]

Article DOI: https://doi.org/10.3201/eid2812.212612

Systematic Review and Meta-analysis of Lyme Disease Data and Seropositivity for *Borrelia burgdorferi*, China, 2005–2020

Appendix

Appendix Table 1. Search strategies and results by database

Database	Search strategy	Results
PubMed	Borreliosis OR lyme OR borrelia<br>Filters: Full text, from 2005/1/1 - 2020/12/31	8,537
EMBASE	(((borrelia AND (2005:2020[pdat])) OR (lyme AND (2005:2020[pdat]))) OR (borreliosis AND (2005:2020[pdat])))<br>Filters: Date: Publication years 2005 – 2020; Pub. Types: Article, Data Papers, Review	10,445
Global Health (CABI Direct)	((borrelia OR lyme OR borreliosis) AND yr:[2005 TO 2020]) AND (((item-type:(("Journal article")))))	6,348
CNKI	Topic: Lyme disease (vague) OR Topic: Borrelia burgdorferi (vague) OR Topic: Lyme disease AND Serum (vague)<br>Publication time: 1st Jan 2005 - 31st Jan 2020	2,032
Wanfang Data	All (vague): Lyme disease; All (vague): Borrelia burgdorferi; All (vague): Lyme disease AND Serum<br>Publication time: 1st Jan 2005 - 31st Jan 2020	3,658

Appendix Table *2*. Studies included in systematic literature review contributing to seropositivity analyses and associated data points

Study	Year(s) of data collection	Province of data collection	Diagnostic test(s)	Antibody*	Sero-positivity, (%)†	Study Denominator	Exposure category	Study population details
Chen et al. (*1*)	2011	Beijing	ELISA	IgG	4.6%	549	Moderate exposure risk	Mountain residents of randomly selected survey sites in Miyun, Beijing, with history of defined field activities within one year. No specific forestry exposure mentioned.
Cui et al. (*2*)	2003–2006	Zhejiang	IFA	IgG	11.9%	932	High exposure risk	Forestry (forest farms) workers and mountain residents in three districts of Hangzhou, Zhejiang.
Dong et al. (*3*)	2004	Neimenggu (Inner Mongolia)	IFA	IgG	1. 10.4%<br>2. 33.3%	1. 671<br>2. 84	1. High exposure risk<br>2. Clinical suspicion	1. Forestry residents from forest farms of Greater Khingan, Neimenggu<br>2. Hospitalized psychiatric patients in the forestry area of Greater Khingan, Neimenggu
Dou et al. (*4*)	2013	Beijing	ELISA + WB	IgG	5.1%	801	Moderate exposure risk	Mountain residents lived near Miyun Reservoir in the northern suburb of Beijing. No specific forestry exposure mentioned.
Du et al. (*5*)	2010	Henan	IFA	IgG	10.3%	126	High exposure risk	Local residents in the mountain regions of Henan with high vegetation coverage and high risk of tick exposure during animal contact.

Study	Year(s) of data collection	Province of data collection	Diagnostic test(s)	Antibody*	Sero-positivity, (%)†	Study Denominator	Exposure category	Study population details
Geng et al. (*6*)	2001–2006	Multiple Provinces	IFA + ELISA	IgG & IgM	16.3%	827	Clinical suspicion	Serum samples from healthcare-seeking patients with clinical suspicion of Lyme disease (unclear forestry exposure)
Geng et al. (*7*)	2007	Jilin	IFA	IgG	10.8%	545	High exposure risk	Forestry residents from the forest region of Changbai and Tonghua counties, Jilin.
Geng et al. (*8*)	2007–2008	Multiple Provinces	IFA + ELISA	IgG & IgM	15.2%	105	Clinical suspicion	Serum samples from healthcare-seeking patients with clinical suspicion of Lyme disease (unclear forestry exposure)
Geng et al. (*9*)	2009	Heilongjiang	IFA	IgG	17.0%	342	Clinical suspicion	Healthcare seeking patients identified from Mudanjiang Forestry Central Hospital.
Gong et al. (*10*)	2002–2004	Zhejiang	1. IFA<br>2. IFA<br>3. ELISA<br>4. ELISA	IgG	1. 0.1%<br>2. 0.0%<br>3. 4.7%<br>4. 9.8%	1. 958<br>2. 162<br>3. 86<br>4. 430	1. High exposure risk<br>2. Clinical suspicion<br>3. Low exposure risk<br>4. High exposure risk	1. Forestry workers, mountain farmers, and other residents in the forestry area of Kaihua county and Tiantai county, Zhejiang<br>2. Suspected Lyme disease cases identified from community health centers. All surveyed individuals were local farmers. No specific forestry exposure mentioned.<br>3. Urban or suburb residents from Lishui, Zhejiang<br>4. Forestry (forest farms) workers and farmers from Lishui, Zhejiang
Gu et al. (*11*)	NA	Heilongjiang	ELISA	IgG	33.9%	498	High exposure risk	Forestry residents from multiple forest farms of Xiao Hinggan Mountains, Heilongjiang
Hao et el (*12*).	NA	Multiple Provinces	IFA	IgG	10.3%	3,669	High exposure risk	Human population in forest areas of 8 provinces in China, covering a wide area of the country from north to south. Investigation sites were chosen based on clear evidence of ticks in the environments. Study population had lived in the forest areas and often worked in the fields.
Ji (*13*)	1. 2013<br>2. 2014	Neimenggu (Inner Mongolia)	1. IFA<br>2. ELISA	IgG	1. 16.9%<br>2. 4.1%	1. 136<br>2. 98	1. High exposure risk<br>2. Low exposure risk	1. Individuals from mountain region, forestry area<br>2. Individuals from plains area
Li et al. (*14*)	2008	Yunnan	ELISA	IgG	2.4%	42	Low exposure risk	University students. A convenient sample taken from health checks in a university in Yunnan. No forestry exposure mentioned.
Li et al. (*15*)	2008–2009	Shaanxi	IFA	IgG & IgM	6.7%	194	High exposure risk	Forestry workers, mountain farmers, and other local residents with long-term residency (greater than 5 years) in three forestry areas of Shaanxi.

Study	Year(s) of data collection	Province of data collection	Diagnostic test(s)	Antibody*	Sero-positivity, (%)†	Study Denominator	Exposure category	Study population details
Li et al. (*16*)	2013	Hainan	1. IFA<br>2. IFA + WB	IgG	1. 12.4%<br>2. 2.8%	1. 251<br>2. 251	Clinical suspicion	Healthcare-seeking patients identified from a hospital in Hainan. Suspected cases were defined as patients with clinical diagnosis of neurological symptoms.
Li et al. (*17*)	2015	Hainan	1. IFA<br>2. IFA + WB	IgG	1. 3.3%<br>2. 1.5%	1. 1,334<br>2. 1,334	Clinical suspicion	Healthcare-seeking patients identified from multiple hospitals in Hainan. Suspected cases were defined as patients with clinical diagnosis of arthritis and/or neurological disease suspected of Lyme disease (excluding rheumatoid arthritis and other related diseases).
Lin (*18*)	NA	Jilin	ELISA	IgG	8.3%	218	High exposure risk	Forestry residents from multiple forest farms of Changbai mountain, Jilin
Lin et al. (*18*)	2007	Fujian	IFA	IgG	17.1%	269	High exposure risk	Forestry workers, mountain farmers, and other local residents of the north forest area (Wuyi Mountain) in Fujian.
Liu et al. (*19*)	2006	Hunan	IFA	IgG	10.2%	914	High exposure risk	Forestry workers, mountain farmers, and other local residents in the mountain forestry areas of Shandong
Liu et al. (*20*)	2010–2011	Heilongjiang	IFA	IgG	18.3%	180	Clinical suspicion	Healthcare-seeking patients with recent tick bite in the past 2 months identified from Mudanjiang Forestry Central Hospital, Heilongjiang. The participants usually presented with tick-borne disease related clinical symptoms.
Long et al. (*21*)	2013–2014	Xinjiang	IFA	IgG	5.7%	637	Low exposure risk	Voluntary blood donors (urban residents) in People's Hospital of Xinjiang Uygur Autonomous Region. Most of the urban residents were not exposed to forestry or pastoral environments.
Song et al. (*22*)	2000–2003	Tianjin	IFA	IgG	1. 6.9%<br>2. 1.8%	1. 735<br>2. 170	1. High exposure risk<br>2. Low exposure risk	1. Local residents from mountain regions or mid-levels of Tianjin<br>2. Local residents from plains areas of Tianjin
Sun et al. (*23*)	1998–2003	Xinjiang	IFA or ELISA	IgG & IgM	13.0%	7,956	Moderate exposure risk	Workers and other local residents of Xinjiang prospecting bureau of Henan oil field, in natural foci of Lyme disease in Xinjiang.
Sun et al. (*24*)	2002–2004	Gansu	IFA	IgG	12.9%	240	High exposure risk	Forestry (forest farms) workers in both protected natural forests and nursey forests.
Tan et al. (*25*)	1999–2004	Xinjiang	1. IFA<br>2. IFA + WB	IgG	1. 12.1%<br>2. 3.1%	1. 223<br>2. 223	High exposure risk	Natural population (including residents and forestry workers) in natural foci of Lyme disease in Southern Mountainous Area of Urumqi, Xinjiang. The survey sites are exposed to high level of forests, pastures, wild animals and plants.

Study	Year(s) of data collection	Province of data collection	Diagnostic test(s)	Antibody*	Sero-positivity, (%)†	Study Denominator	Exposure category	Study population details
Tan et al. (*26*)	2000–2004	Xinjiang	IFA + WB	IgG	NA‡	NA‡	1. High exposure risk<br>2. Moderate Exposure Risk	1. Natural population in natural foci of Lyme disease in six districts of Xinjiang, including forestry workers, pastoral workers, and border soldiers.<br>Individuals with no clinical suspicion of Lyme disease were randomly surveyed from hospitals and out-patient clinics of Xinjiang as comparison group. No specific forestry exposure mentioned but noted this region in Xinjiang is endemic to Lyme disease.
Tan et al. (*27*)	1. 2002<br>2. 2006	Xinjiang	WB	IgG	1. 0.3%<br>2. 37.0%	1. 1,406<br>2. 119	1. Moderate exposure risk<br>2. Moderate exposure risk	1. Human population in natural foci of Lyme disease in Xinjiang were selected.<br>2. Individuals in natural foci of Lyme disease in Xinjiang with negative test results in the previous epidemiologic study in 2002.
Wang et al. (*28*)	1990	Shandong	IFA	IgG	6.3%	1,934	High exposure risk	Forestry (forest farms) workers and local residents of multiple regions in Shandong.
Wang et al. (*28*)	1990–2003	Guizhou	IFA	IgG	5.0%	139	Moderate exposure risk	Workers and other residents (including mostly agrarian population and selective forestry workers) living in agricultural county.
Wang et al. (*29*)	2005	Beijing	IFA	IgG	1. 9.5%<br>2. 3.0%<br>3. 8.5%	1. 370<br>2. 331<br>3. 47	1. Moderate exposure risk<br>2. Low exposure risk<br>3. Clinical suspicion	1. Local residents and pastoral workers in the natural foci of Lyme disease in Miyun district, Beijing. No forestry exposure mentioned.<br>2. Local residents engaged in tourism reception work at home. Had low or minimal risk of tick exposure.<br>3. Local residents with neurologic disorders identified from local mental health care facility.
Wang et al. (*30*)	2006	Jilin	IFA	IgG	6.6%	617	Moderate exposure risk	The mountainous area and the border area in conjunction with North Korea. Survey sites in Tonghua, Ji'an, and Changbai counties of Jilin are selected, which are representative of Lyme disease-endemic area in Jilin. No specific forestry exposure mentioned.
Wang et al. (*31*)	2003–2008	Jilin	IFA	IgG	5.0%	909	Low exposure risk	Local residents from the plains regions of five districts in Jilin
Wang et al. (*31*)	NA	Jilin	ELISA	IgG	7.0%	1,002	High exposure risk	Local residents from the mountainous forest region of Jilin county and Yanbian county, Jilin. These regions have high forestry coverage

Study	Year(s) of data collection	Province of data collection	Diagnostic test(s)	Antibody *	Sero-positivity, (%)†	Study Denominator	Exposure category	Study population details
								and Lyme disease-endemic area.
Wu et al. (*32*)	2016	Xinjiang	ELISA	IgG	4.1%	1,500	Low exposure risk	Individuals participated in health checks in the urban region of southern Xinjiang. No forestry exposure mentioned.
Xia et al. (*33*)	NA	Jilin	ELISA	IgG	6.8%	281	High exposure risk	Forestry residents from multiple forest farms of Tonghua county, Jilin.
Xie et al. (*34*)	NA	Guangdong	IFA or ELISA	IgG	1. 10.3%<br>2. 10.3%	1. 1,191<br>2. 993	1. High exposure risk<br>2. Moderate exposure risk	1. Natural population in living in forestry area (forest farms) of Meizhou, Guangdong (natural foci of Lyme disease).<br>2. Natural population living in non-forestry area of Meizhou, Guangdong (natural foci of Lyme disease).
Yang et al. (*35*)	2016	Beijing	ELISA + WB	IgG	9.3%	140	Clinical suspicion	Healthcare-seeking patients identified from 10 community health centers in Lyme disease natural foci of Miyun, Beijing. Suspected cases were defined as patients with clinical diagnosis of arthritis (excluding rheumatoid arthritis and other related diseases).
Ye et al. (*36*)	2005	Fujian	IFA	IgG	2.1%	239	Low exposure risk	Individuals participated in health checks (including pregnant women and drug users) in the urban region of Xiamen, Fujian. No specific forestry exposure mentioned.
Yu et al. (*37*)	2006	Gansu	IFA	IgG	10.9%	522	High exposure risk	Forestry (forest farms) workers and local residents of Diebu county, Gansu.
Yue & Shi (*38*)	NA	Qinghai	IFA	IgG	15.1%	1,108	High exposure risk	Forestry workers, farmers, and other forestry residents in agricultural or pastoral county.
Zhang et al. (*39*)	2005	Shanxi	IFA	IgG	6.2%	227	High exposure risk	Forestry workers and mountain residents in Shanxi
Zhang et al. (*40*)	2009	Multiple Provinces	IFA	IgG	7.0%	725	High exposure risk	Forestry residents of four provinces in northern China, including Xinjiang, Gansu, Ningxia, and Shaanxi
Zhang et al. (*41*)	2013	Hainan	1. IFA<br>2. IFA+ WB	1. IgG & IgM<br>2. IgG & IgM	1. 16.6%<br>2. 2.3%	1. 259<br>2. 259	Clinical suspicion	Healthcare-seeking patients identified from a hospital in Hainan. Suspected cases were defined as patients with clinical diagnosis of arthritis or neurological disease suspected of Lyme disease.
Zhao et al. (*42*)	2012	Xinjiang	ELISA	IgG	1. 5.9%<br>2. 2.9%	1. 101<br>2. 34	1. High exposure risk<br>2. Moderate exposure risk	1. Border guard soldiers stationed in mountain/forest habitats in northern Xinjiang.<br>2. Border guard soldiers stationed in the Gobi/desert habitats in northern Xinjiang. Indicated occupational risk of tick exposure.
Zhu et al. (*41*)	2013	Hainan	1. IFA<br>2. IFA+ WB	IgG	1. 7.7%<br>2. 1.3%	1. 542<br>2. 542	Clinical suspicion	Healthcare-seeking patients identified from a hospital in Hainan. Suspected cases were defined as patients with

Study	Year(s) of data collection	Province of data collection	Diagnostic test(s)	Antibody*	Sero-positivity, (%)†	Study Denominator	Exposure category	Study population details
Zhu et al. (*43*)	2015	Hainan	1. IFA 2. IFA+ WB	IgG	1. 3.4% 2. 1.4%	1. 900 2. 900	Clinical suspicion	clinical diagnosis of arthritis or neurological disease suspected with B. burgdorferi infection (excluding related diseases). Healthcare-seeking patients identified from two hospitals in western region, Hainan. Suspected cases were defined as patients with clinical diagnosis of arthritis or neurological disease suspected of Lyme disease (excluding rheumatoid arthritis and other related diseases).
Zhuang et al. (*44*)	2006	Guizhou	1. IFA 2. IFA+ WB	IgG	1. 5.3% 2. 1.1%	1. 1,233 2. 1,233	Low exposure risk	Rural population of 8 counties in Guizhou (alpine, mainly plains area). No specific forestry exposure mentioned.

*Antibody measured reflects data used for analysis. For IgG and IgM, results were presented collectively without separation. ELISA, enzyme-linked immunosorbent assay; IFA, immunofluorescence assay; IgG, immunoglobulin G; IgM, immunoglobulin M; WB, Western immunoblot.
†Multiple estimates from same source presented as the total estimate.
‡Multiple data points available in Supplementary Table 1.

References

1. Chen YL, Yang YS, Jia LL, Geng LB, Wang YQ, Zheng FH. Seroprevalence of Lyme disease in Miyun county of Beijing, China. Can J Public Health. 2016;10:83–5.

2. Cui WW, Deng J, Shi SF, Huang RJ, Xu K, Huang CX, et al. 北京市密云县莱姆病血清流行病学调查. Chin J Prev Med. 2007;8:382–6.

3. Dong JH, Zhu JH, Yin FR. 大兴安岭林区莱姆病人群患病状况调查. Pract Prev Med. 2007;14:1457–8.

4. Dou X, Lyu Y, Jiang Y, Tian L, Li X, Lin C, et al. Seroprevalence of Lyme disease and associated risk factors in rural population of Beijing. Int J Clin Exp Med. 2015;8:7995–9. PubMed

5. Du YH, Zhao JY, Lu X, Bai JM, Xia SL. Serological epidemiology investigation of Lyme disease in Henan Province [in Chinese]. Xian Dai Yu Fang Yi Xue. 2012;39:3681–2.

6. Geng Z, Hou XX, Hao Q, Hu GL, Wan KL. Anti-borrelia burgdorferi antibody response in 827 patients with suspected Lyme disease [in Chinese]. Zhongguo Meijie Shengwuxue Ji Kongzhi Zazhi. 2007;18:219–21.

7. Geng Z, Hou X-X, Guo JH, Huang X, Wang CS, Wang B, et al. Epidemiological investigation on Lyme disease in Changbai and Tonghua county, Jilin province [in Chinese]. Zhongguo Meijie Shengwuxue Ji Kongzhi Zazhi. 2010;21:572–5.

8. Geng Z, Hou X-X, Wan K-L, Hao Q. *Borrelia burgdorferi* antibody levels in 105 patients with suspected Lyme disease [[in Chinese]. Zhongguo Meijie Shengwuxue Ji Kongzhi Zazhi. 2010;21:65–7.

9. Geng Z, Huo QB, Hou XX, Hao Q, Wan KL. Analysis on the result of detecting serum from 342 patients with suspected Lyme disease in Mudanjiang forestry area [in Chinese]. Guide of China Medicine. 2010;8:182–4.

10. Gong ZY, Jiang LP, Wang Z, Gao Y, Fang CF, Wang W, et al. Serological epidemiology investigation of Lyme disease in Zhejiang Province [in Chinese]. Ji Bing Jian Ce. 2005;20:510–2.

11. Gu CG, Cao XH, Jia YP, Yang SJ, Kong JS. Seroepidemiological investigation of Lyme disease in a part of forest region of Xiao Hinggan mountains [in Chinese]. Xian Dai Yu Fang Yi Xue. 2014;41:1125–9.

12. Hao Q, Geng Z, Hou XX, Tian Z, Yang XJ, Jiang WJ, et al. Seroepidemiological investigation of lyme disease and human granulocytic anaplasmosis among people living in forest areas of eight provinces in China. Biomed Environ Sci. 2013;26:185–9. PubMed

13. Ji CL. 宝格**达山阿拉坦合力地区蜱**传莱姆病疫源地调查. Lin Chuang Yi Xue Za Zhi. 2017;4:8886–8.

14. Li J, Tong SF, Yang ZF, He RY, Cui YH, Zhu ZW, et al. Lyme disease and Scrub Typhus serosurvey among college freshmen in Yunnan Province [in Chinese]. Journal of Kunming Medical University. 2014;35:21–3.

15. Li ZQ, Gong ZW, Fei JX, Shi SG, Liu T, Feng XY, et al. 陕西省部分林区莱姆病血清流行病学调查研究. Chin J Zoonoses. 2010;26:879–80.

16. Li S, Chen T, Li H, Yu SY, Chen H, Zhu X. 三亚地区神经症状患者莱姆病血清筛查分析. Jianyan Yixue Yu Linchuang. 2018;15:1465–7.

17. Li S, Zhang L, Li H, Hou XX, Chen T, Miao GQ, et al. Test results for detection of antibody against Lyme disease in certain patients in the northeast of Hainan province. Pract Prev Med. 2020;27:1068–71.

18. Lin CH. 长白山林区莱姆病的血清学调查. Journal of Medical Science Yanbian University. 2008;31:272–4.

19. Liu FQ, Hao Q, Gao LD, Geng Z, Zhan ZF, Hou XX, et al. Preliminary epidemiological investigation of lyme disease in Hunan province [in Chinese]. Ji Bing Jian Ce. 2008;23:337–40.

20. Liu HB, Wei R, Ni XB, Zheng YC, Huo QB, Jiang BG, et al. The prevalence and clinical characteristics of tick-borne diseases at One Sentinel Hospital in Northeastern China. Parasitology. 2019;146:161–7. PubMed https://doi.org/10.1017/S0031182018001178

21. Long J, Niu XS, Wen J, Mao WJ, Zhong T, Li HY, et al. The survey of *Borrelia burgdorferi* infection among blood donors in Urumqi area [in Chinese.]Zhongguo Meijie Shengwuxue Ji Kongzhi Zazhi. 2017;28:280–2.

22. Song CY, Liu H, Chen JY, Yu FT, Zhao ZW, Jia YH, et al. Epidemic of Lyme disease research in Jixian county of Tianjin [in Chinese]. Xian Dai Yu Fang Yi Xue. 2012;39:5419–21.

23. Sun HS, Tian Z, Geng Z, Hou XX, Hao Q, Jiang Y, et al. An investigation on Lyme disease in Xinjiang Prospecting Bureau of Henan Oilfield [in Chinese]. Zhongguo Meijie Shengwuxue Ji Kongzhi Zazhi. 2005;16:209–11.

24. Sun Y, Liu ZJ, Xu RM, Shi SZ, Zhao TY. Effects of natural forests protection on the epidemical characteristics of Lyme disease. Acta Parasitol Med. Entomol Sin. 2005;12:106–11.

25. Tan YH, Liu Y, Sun H, Yu LH, Mangu N, Zhu L, et al. Surveillance and analysis on the natural foci of Lyme disease in Southern Mountainous Area of Urumqi, Xinjiang [in Chinese]. Zhongguo Meijie Shengwuxue Ji Kongzhi Zazhi. 2011;22:141–3.

26. Tan YH, Liu Y, Sun H, Yu LH, Long J, Niu XS, et al. Surveillance of Lyme disease in Xinjiang Uygur autonomous region during 2000-2004. J Clin Neurosci. 2007;15:158–61.

27. Tan YH, Liu Y, Wan KL, Hao Q, Sun H, Yu LH, et al. Molecular epidemiological study of *Borrelia burgdorferi* infection among population in Xinjiang, China [in Chinese]. Zhongguo Meijie Shengwuxue Ji Kongzhi Zazhi. 2013;24:297–300.

28. Wang LJ, Hou XX, Chen ZL, Hao Q, Tao XR, Wan KL. Seroepidemiological study of Lyme disease in Shandong province [in Chinese]. Zhongguo Meijie Shengwuxue Ji Kongzhi Zazhi. 2007;18:306–7.

29. Wang HY, Hou XX, Li LQ, Geng Z, Wang QY, Hao Q. Seroepidemological investigation of lyme disease in Miyun country, Beijing in 2005. Chin Prev Med. 2009;10:737–9.

30. Wang CS, Wan KL, Guo JH, Geng Z, Wang B, Chen DG, et al. 吉林省边境地区莱姆病流行状况调查. Gong gong wei sheng yu yu fang yi xue. 2008;19:48–9.

31. Wang CS, Wan KL, Yang XJ, Wang B, Zhang GF, Liang XP, et al. 吉林省平原地区莱姆病流行病学调查. Pract Prev Med. 2009;16:341–2.

32. Wu JY, Gong TM, Wang L, Jiang W, Ke Y. Investigation on serum epidemiology of tick-born Lyme disease in different population in Kashi city of Xinjiang (新疆喀什市不同人群蜱传莱姆病血清流行病学调查). J Trop Med. 2017;17:1663–5.

33. Xia QB, Wang CS, Li YX, Dai JL, Chen DG, Ge YH, et al. Investigation and laboratory analysis of Lyme disease in Tonghua. Chinese Journal of Health Laboratory Technology. 2007;17:1857–8.

34. Xie LC, Wan KL, Guo Y, Xu SE, Zhang ZF, Pan LX, et al. Study of the natural foci of Lyme disease in the City of Meizhou, Guangdong Province [in Chinese]. Zhongguo Bingyuan Shengwuxue Zazhi. 2009;4:575–8.

35. Yang YS, Lyu YN, Chen YL. Serological investigation in arthritis patients with Lyme disease in Miyun district, Beijing [in Chinese]. Zhongguo Meijie Shengwuxue Ji Kongzhi Zazhi. 2017;28:490–1.

36. Ye X, Wang RF, Li GW. An investigation on infection of Lyme disease in person and tick vectors in Xiamen (厦门地区部分人群莱姆病感染及蜱媒调查). J Prev Med Inf. 2007;23:282–3.

37. Yu DS, Geng Z, Jiang JX, Hao Q, Chen JH, Wang P. Investigation on the natural focus of Lyme disease in Diebu county of Gansu province [in Chinese]. Zhongguo Meijie Shengwuxue Ji Kongzhi Zazhi. 2009;20:57–8.

38. Yue J-N, Shi Y. Epidemiological investigation of Lyme disease in parts of forest areas in Qinghai province [in Chinese]. Zhongguo Meijie Shengwuxue Ji Kongzhi Zazhi. 2009;20:358–9.

39. Zhang JM, Yang HJ, Li XM, Gao XF, Yang T, Zhao ZS, et al. Investigate the infection of Lyme disease in Yuanqu county [in Chinese]. Zhongguo Yaowu Yu Linchuang. 2006;6:490–1.

40. Zhang F, Zhong ZH, Gong ZW, Zhang JJ, Liu HF, Xiao YS, et al. Study on the sero-epidemiology of Lyme disease from certain areas of northwestern China [in Chinese]. Zhonghua Liu Xing Bing Xue Za Zhi. 2009;30:1318–9.

41. Zhang L, Zhu X, Hou X, Geng Z, Chen H, Hao Q. Test of 259 serums from patients with arthritis or neurological symptoms confirmed existence of Lyme disease in Hainan province, China. Int J Clin Exp Med. 2015;8:9531–6. PubMed

42. Zhao Y, Liu R, Zhang GL, Liu XM, Sun X, Zheng Z, et al. 新疆北部某边防部队蜱传疾病血清流行病学调查. J Prev Med China PLA. 2014;32:324–5.

43. Zhu X, Hou XX, Yu L, Zhang L, Chen YY, Miao GQ, et al. Serological investigation on Lyme disease in Western Region of Hainan Province. Chin J Zoonoses. 2020;36:313–7.

44. Zhuang Y, Wang DM, Jiang WJ, Zhou JZ, Hu J, Yu C. 贵州省2006 年农村人群感染莱姆病状况调查. Guizhou Medicine. 2009;33:169–70. PubMed
